# The status quo of digital transformation in insurance sales: an empirical analysis of the german insurance industry

**DOI:** 10.1007/s12297-021-00507-y

**Published:** 2021-11-29

**Authors:** Christian Eckert, Johanna Eckert, Armin Zitzmann

**Affiliations:** 1 Coburg University of Applied Sciences and Arts, Friedrich-Streib-Str. 2, 96450 Coburg, Germany; 2 School of Business, Economics and Society, Chair of Insurance Economics and Risk Management, Friedrich-Alexander University Erlangen-Nürnberg (FAU), Lange Gasse 20, 90403 Nürnberg, Germany; 3 NÜRNBERGER Versicherung, Ostendstr. 100, 90334 Nuremberg, Germany

**Keywords:** Digital transformation, Empirical study, German insurance industry, Insurance sales, Survey

## Abstract

In this paper, we focus on the impact of digital transformation on traditional ways to sell insurance products. Our goal is to investigate how widespread the use of digital technologies in insurance sales is and which values of these digital technologies are perceived by insurance intermediaries. Moreover, we aim to analyze underlying influencing factors in this regard. We conducted a survey in July 2020, i.e., after the first wave of COVID-19, in which 671 exclusive agents from various insurance companies, independent agents and independent brokers from Germany participated. Our results show that even at this point of time, in a high proportion of sales workforces, digital transformation is not yet very advanced. Further analyses show that exclusive agents and younger people are further ahead in digital transformation even though COVID-19 pushes digital life across all ages and social classes. Based on our results, we derive initial recommendations for action for the insurer.

## Abstract (de)

In diesem Artikel fokussieren wir uns auf die Auswirkungen der digitalen Transformation auf die traditionellen Vertriebswege in der Versicherungsbranche. Unser Ziel ist es zu untersuchen, wie verbreitet der Einsatz digitaler Technologien im Versicherungsvertrieb ist und welchen Wert die Vermittler diesen digitalen Technologien zuschreiben. Darüber hinaus wollen wir die zugrunde liegenden Einflussfaktoren in diesem Zusammenhang analysieren. Wir haben im Juli 2020, d. h. nach der ersten COVID-19-Welle, eine Umfrage durchgeführt, an der 671 gebundene Versicherungsvertreter verschiedener Versicherungsunternehmen, Mehrfachvertreter und Versicherungsmakler aus Deutschland teilnahmen. Unsere Ergebnisse zeigen, dass die digitale Transformation auch zu diesem Zeitpunkt bei einem hohen Anteil der Vertriebsmitarbeiter noch nicht weit fortgeschritten ist. Weitere Analysen zeigen, dass gebundene Versicherungsvertreter und jüngere Menschen Vorreiter bei der digitalen Transformation sind, obwohl COVID-19 das digitale Leben über alle Altersgruppen und Gesellschaftsschichten hinweg beschleunigt. Basierend auf unseren Ergebnissen leiten wir erste Handlungsempfehlungen für Versicherer ab.

## Introduction

Like most other industries, the insurance industry is strongly affected by digital transformation (see Eling and Lehmann 2018; Eckert and Osterrieder 2020). In an age of customer centricity in which companies like Amazon set new standards, digital transformation is not an end in itself but a means to meet changed customer expectations. There are different definitions of digital transformation and digitalization in scientific and practical literature, e.g., Ingleton et al. (2011), Hiendlmeier and Herting (2015), Tischhauser et al. (2016) and Eling and Lehmann (2018). For this article, we follow Eling and Lehmann (2018), who understand digital transformation to refer to the integration of the analogue and digital worlds using new technologies, which improve customer interaction, data availability and business processes. We are thus referring exclusively to the economic consequences of digital transformation.

According to Eling and Lehmann (2018), digital transformation affects the whole value chain of insurance companies. For instance, it allows the automation of business processes and decisions. Moreover, existing products are being changed, e.g., telematics in motor vehicle insurance and new products are being created, e.g., cyber risk insurance. Finally, the way insurance companies interact with their customers is also fundamentally changing, which is why insurance sales are particularly affected.

In insurance sales, we refer to the definition of insurance distribution of the Revised Directive (EU) 2016/97 on Insurance Distribution (IDD) (European Union 2016). This states that “‘insurance distribution’ means the activities of advising on, proposing, or carrying out other work preparatory to the conclusion of contracts of insurance, of concluding such contracts, or of assisting in the administration and performance of such contracts, in particular in the event of a claim, including the provision of information concerning one or more insurance contracts in accordance with criteria selected by customers through a website or other media and the compilation of an insurance product ranking list, including price and product comparison, or a discount on the price of an insurance contract, when the customer is able to directly or indirectly conclude an insurance contract using a website or other media.”

According to the definition above, a distinction can be made between three steps, whereby we additionally locate customer acquisition in the field of sales:

0. Customer acquisition: Acquiring new customers.
1. Advice: Information and advice.
2. Conclusion: Proposing and preparing for the conclusion of an insurance contract as well as the conclusion of contracts.
3. Support: Participation in administration and fulfillment (after-sales, maintaining contacts, processing customer concerns and support in the event of a claim).

Note that classical definitions of (insurance) sales typically do not include step three (see, e.g., Beenken 2019). For example, while cross- and up-selling is part of the sales process, after-sales is not. However, since we aim to investigate the status quo of insurance intermediaries regarding digital transformation and step three is also part of their everyday business, we follow the definition of the European Union (2016) and also consider step three ‘Support.’

Traditionally, to receive information about insurance products, customers needed personal contact with insurance intermediaries. Nowadays, a lot of information about products is available online and many products can be purchased directly online without personal interaction. Hence, digital transformation enables new sales channels (e-commerce), but traditional sales channels also benefit, e.g., via messenger services or video chats for customer interaction. Digital transformation is able to offer the insurance agents and brokers opportunities to better fulfill their core task as a service provider for the customer. There is potential both in the interaction with customers (e.g., transparent and target-oriented advice, faster processes) and in the context of efficiency gains in other activities (e.g., time gain in administrative activities), which in turn allows the agents and brokers to spend more time with their customers. In this way, the insurance intermediaries might be able to meet today’s changing customer expectations. In particular, in retirement planning young people often have no impetus to forego consumption today in order to secure an income in old age (see Zitzmann 2018). With personal advice, appropriate education can be provided and gentle nudging can lead to adequate retirement provision (see Zitzmann 2018). Moreover, the millennials study by NÜRNBERGER Versicherung and Frankfurt Business Media (2018) shows that millennials, i.e., even digital natives, want personal advice on retirement provision and risk coverage. In doing so, however, they demand understandable explanations and individual communication channels. Insurance companies, agents and brokers are facing these increased requirements, but also opportunities.

The goal of our paper is to investigate the status quo of digital transformation in traditional insurance sales in the German insurance industry. We aim to study how widespread the use of certain digital applications in traditional insurance sales is and investigate the perceived benefit from these digital applications. Moreover, we analyze underlying influencing factors regarding digital transformation. For this purpose, we conducted a survey in July 2020, in which 671 German exclusive agents from various insurance companies, independent agents and independent brokers participated.^1^ Since the first nationwide lockdown in Germany due to COVID-19 started on March 23, 2020 and ended with greater easing of restrictions in early May 2020, we conducted our survey after the first wave of the COVID-19 crisis.

Our results show that even after the first wave of the COVID-19 crisis, which means that for several weeks selling insurance products was not possible via face-to-face meetings, a high proportion of sales groups or teams within the insurance industry (hereafter referred to as ‘sales units’) hardly use digital applications. For instance, about half of the sales units do not use video chats and see little benefit in doing so, although remote advisory services help to maintain customer relationships in these times. Among the digital applications, the insurance intermediaries appreciate the benefit from digitized processes, particularly in the context of application and conclusion of contracts or claims management. Carrying out further studies, we find that exclusive agents are more advanced in digital transformation than independent agents and brokers, which is the same for sales units made up of younger members compared with older members. Although the COVID-19 crisis pushes digital life across all ages and social classes, these differences are statistically significant. In addition to a more widespread use of digital applications, these subgroups also perceive a greater value added by digital transformation.

The reminder of this paper is structured as follows. Sect. 2 provides a theoretical background regarding digital transformation in insurance sales, which allows us to derive the survey questionnaire and our hypotheses. In Sect. 3, we explain our research methodology. Sect. 4 presents our results, first, descriptive statistics of the survey and second, the results regarding our hypotheses. Sect. 5 derives recommendations for action and discusses limitations as well as further research. Finally, Sect. 6 offers the conclusion.

## Theoretical background and hypotheses development

First, we discuss relevant digital technologies, based on which we identify potential digital applications in insurance sales. Analyses by Eckert and Osterrieder (2020) and Eling and Lehmann (2018) show that big data, artificial intelligence, cloud computing and new communication channels are strong drivers of digitalization.

*Big data* refers to data sets that meet certain characteristics. Big data are usually characterized by the ‘three Vs’: volume, velocity and variety (Owadally et al. 2019; Eckert and Osterrieder 2020). There are some extensions within the multitude of definitions. For example, Ravi and Kamaruddin (2017) argue that big data are also characterized by veracity and value, i.e., ‘five Vs’.

There is also no unique definition for *artificial intelligence*, but it is often understood as the ability of machines to master certain cognitive functions, such as learning from experience or solving problems (Russell and Norvig 2009). Machine learning is an important sub-category of artificial intelligence (Kelley et al. 2018; Eckert and Eckert 2020); in contrast to traditional algorithms, which generate an output from a given input using predetermined transformation rules, machine learning allows algorithms to learn the transformation rules independently based on given inputs and outputs. For this purpose, the algorithms reduce the deviations between the generated and the desired output within an iterative training process.

*Cloud computing* enables location-independent, convenient and demand-controlled network access to a shared pool of configurable computing resources (Bundesanstalt für Finanzdienstleistungsaufsicht (BaFin; the German Federal Financial Supervisory Authority) 2018; European Insurance and Occupational Pensions Authority (EIOPA) 2020). A distinction can be made between three service models for the procurement of cloud services: Software as a Service (SaaS), Platform as a Service (PaaS) and Infrastructure as a Service (IaaS) (BaFin 2018). There are four types of provision of cloud computing: private clouds, community clouds, public clouds and hybrid clouds (EIOPA 2020).

While the above technologies can be used in particular for data acquisition and analysis, and also for data storage, digital transformation is fundamentally changing the way people communicate with one another (*new communication channels*). People share information via e‑mail, web pages, social networks, messengers, video calls and video platforms. Smartphones and tablets with corresponding apps are replacing traditional desktop computers and robotics offers new ways for communication, e.g., chatbots in customer service or robo-advisors in automated asset management (Eling and Lehmann 2018).

Table 1 provides an overview of potential digital applications for insurance sales. Based on the definition of ‘insurance distribution’ by the European Union (2016), we consider four sales process steps: customer acquisition, advice, conclusion and support. For each sales process step, Table 1 presents applications using digital technologies. For instance, in customer acquisition insurance intermediaries can leverage customer relationship management (CRM) systems regarding cross- and up-selling applying big data, artificial intelligence and cloud computing. Table 1 shows that in particular, CRM systems, omnichannel customer interaction and digital interfaces to insurance companies are possible applications along the sales process. The main goal of these applications is to support the sales forces in enhancing the customer experience.

**Table 1:** Applications of digital technologies in insurance sales

Sales process step	Digital application	Used digital technologies
Customer acquisition	Customer relationship management system (CRM system): Structured collection and analysis of customer contacts and information – Can be enriched from and linked to new data sources: e‑mails, phone calls, social networks, e.g., use of social media listening platforms to proactively address customers at life events – Automated portfolio analysis: automatically generated advice and situation-related recommendations regarding, e.g., up- and cross-selling, customer lifetime value calculation, churn management	Big data Artificial intelligence Cloud Computing
Advice	Omnichannel customer interaction: Personal contact (face-to-face), telephone, e‑mail, mail, messenger services, video chat/sharing of screen views, e.g., co-browsing	New communication channels Cloud Computing
	Digital consulting software, e.g., on a tablet	New communication channels
	CRM system (see above)	Big data Artificial intelligence Cloud Computing
Conclusion	Smart Underwriting: – Risk assessment at the point of sale – Fraud detection when signing a contract	Artificial intelligence
	Digital interface with insurance company: – Digital signature/signature pad – Contract can be transmitted digitally to insurance company	Artificial intelligence
Support	Omnichannel customer interaction (see above)	New communication channels
	CRM system (see above)	Big data Artificial intelligence Cloud Computing
	Digital interface with insurance company: – Digital signature/signature pad – Digital exchange regarding business transactions	Artificial intelligence

Based on the digital applications presented in Table 1, we designed our questionnaire, which we will describe in more detail in Sect. 3. With our survey, we aim to analyze the status quo of the usage of these applications in insurance sales and how much value they are able to add. In addition, we aim to dive deeper and investigate influencing factors on the status quo of digital transformation in insurance sales and attitudes towards digital transformation.

Compared with independent agents and independent brokers, the connection of exclusive agents with an insurance company is closer. Exclusive agents are solely tied to a specific insurer and do not act as a representative for any other company. Digital experiences in other industries have raised customer expectations in insurance. COVID-19 accelerated this development (Puttaiah et al. 2020) and insurance companies are speeding up their digital transformation. Since agents and brokers are important intermediaries to the customer, insurance companies are very interested in meeting customer expectations to optimize customer journeys and improve customer satisfaction. As insurance companies can apply pressure on their exclusive agents and equip them with the necessary digital infrastructure, we assume that exclusive agents are further ahead in their digital transformation. We state our first hypothesis as follows:

### *Null Hypothesis 1:*

*The extent of the use of digital applications among exclusive agents is the same as among independent agents and brokers*.

Rejecting this hypothesis means that exclusive agents do not use the same number of digital applications as independent agents and brokers.

Furthermore, the subgroup with more experience regarding digital transformation might also appreciate more the benefits from digital applications. This leads to our second hypothesis:

### *Null Hypothesis 2:*

*Exclusive agents value the benefits from digital applications in the same way as independent agents and brokers.*

Rejecting this hypothesis means that exclusive agents appreciate digital applications more or less than independent agents and brokers.

Younger people are typically more open to digital transformation than older people (Avramakis et al. 2020). Consequently, we distinguish between sales units with an average age under 40 years and sales units with an average age exceeding 50 years. However, during the COVID-19 pandemic, digital adoption spread across all generations, e.g., in communication and commerce (Puttaiah et al. 2020), potentially implying diminishing differences between generations. To get more insight in the relationship between age and digital transformation, we formulate our third hypothesis as follows:

### *Null Hypothesis 3:*

*The extent of the use of digital applications among sales units with an average age under 40 years is the same as the extent of the use among sales units with an average age exceeding 50 years.*

Rejecting this hypothesis means that there is a different extent of the use of digital applications among sales units with an average age under 40 years compared with sales units with an average age exceeding 50 years.

Again, we analyze the digital mindset, particularly based on a higher enthusiasm for digital technologies, and state hypothesis four:

### *Null Hypothesis 4:*

*Sales units with an average age under 40 years value the benefits from digital applications in the same way as sales units with an average age exceeding 50 years.*

Rejecting this hypothesis means that sales units with an average age under 40 years appreciate digital technologies more or less than sales units with an average age exceeding 50 years.

## Research methodology

Supported by AssCompact (a multimedia communication platform for the insurance, pension and financial sectors) and the Bundesverband Deutscher Versicherungskaufleute (BVK; the Federal Association of German Insurance Merchants), we carried out a computer-based web survey with single-choice questions in July 2020. Our questionnaire was based on our findings from Sect. 2. A total of 671 participants took part, all of whom sell insurance products. Pretests were conducted with AssCompact and representatives of insurance sales.

In the first part of the questionnaire, we asked for information about the sales unit of the participant, e.g., business type and age structure, and the position of the participant in their unit. The main part of the questionnaire asked about certain applications in the field of digital technologies based on Table 1 (e.g., CRM system, messenger services and video chats), whether the respective sales unit uses this application or at least plans to do so in the future (‘Yes’, ‘No, however, I plan to do so’, ‘No, and currently not planned’, ‘Unknown’). Regarding business transactions in the process steps application of a contract, conclusion of a contract and claims management, we asked for the percentage of business transactions being sent digitally to the insurance company (interval-scaled, 0–100%). Moreover, we asked in each of these cases about the assessment of the (potential) value added by the application. Here, we measured the participant’s perceptions on a seven-point Likert scale (1 = ‘very low’, 7 = ‘very high’).

To test the statistical significance of our Null Hypotheses 1 and 3, we follow previous literature, e.g., Salkind (2010), by applying a chi-squared test and, for the interval-scaled variables regarding digital business transactions, a Welch’s t‑test, respectively. For each chi-squared test, the requirement holds that all expected cell frequencies in the bivariate tabular analysis were (by far) greater than five. Given inequality in variance of the two samples (significance in Levene’s test), we finally choose a modified t‑test, i.e., the Welch’s t‑test.

To test the statistical significance of our Null Hypotheses 2 and 4, we apply the Mann-Whitney U test (Mann and Whitney 1947; Salkind 2010). Since the shapes of the distributions differ between both respective groups based on the Kolmogorov-Smirnov test, we cannot interpret the Mann-Whitney U test as a test of medians, but rather, as a test of stochastic ordering of the samples (Kolmogorov 1933; Mann and Whitney 1947; Smirnov 1948; Hart 2001; Divine et al. 2018).

Note that a limitation of our research methodology might be a selection bias due to using a web survey design. Participants with lower levels of digital affinity might be under-represented because they use the internet less extensively and, therefore, are less likely to participate. Participants with higher levels of digital affinity are more likely to select themselves for the survey (Bethlehem 2010).

## Results

### Descriptive statistics

Tables 2, 3, 4 and 6 show descriptive statistics of our survey with 671 participants. Table 2 shows that a similar number of exclusive agencies (348 in total) and independent agencies and independent brokers (312 in total) took part. We see that in a lot of sales units the average age of the sales force is older than 50 years (307 in total).

**Table 2:** Descriptive statistics regarding the sales units

Variable	Value	Absolute frequency	Relative frequency in %
Type of the company	Exclusive agency	348	46.5
	Independent agency and broker	312	51.9
	Other	11	1.6
Average age of the sales force	Younger than 40 years	127	18.9
	Between 40 and 50 years	193	28.8
	Older than 50 years	307	45.8
	Unknown	22	3.3

**Table 3:** Descriptive statistics regarding the use of digital applications by the sales units (absolute frequency and relative frequency in % in brackets)

Digital application	Do you use this digital application?			
	Yes	No, however, I plan to do so	No, and currently not planned	Unknown
CRM system	495 (73.8)	38 (5.7)	123 (18.3)	15 (2.2)
CRM system capable of working with unstructured data	299 (60.5)	59 (11.9)	95 (19.2)	42 (8.5)
CRM system capable to generate automated recommendations based on portfolio analyses	316 (63.8)	75 (16.1)	80 (15.2)	24 (4.9)
Messenger services for customer interaction	414 (61.7)	44 (6.6)	207 (30.8)	6 (0.9)
Video chat for customer interaction	340 (50.7)	154 (23.0)	174 (25.9)	3 (0.4)
Extended video chats including screen/whiteboard sharing	335 (49.9)	154 (23.0)	168 (25.0)	14 (2.1)
Risk assessment at point of sale with a reduced number of questions	279 (41.6)	110 (16.4)	243 (36.2)	39 (5.8)

**Table 4:** Descriptive statistics regarding the percentage of business transactions in the steps application of a contract, conclusion of a contract and claims management being sent digitally to the insurance company

	Digitally sent business transactions to the insurance company in % in the step …					
Digital application	*N*	Mean	Median	Standard deviation	Minimum	Maximum
Application of a contract	671	77.3	90	27.7	0	100
Conclusion of a contract	671	77.9	90	26.6	0	100
Claims management	671	76.8	90	28.4	0	100

Table 3 presents the descriptive statistics regarding the use of digital applications by the sales units. For each digital application, for instance, messenger services or video chat, we asked the participant whether the sales unit uses this application and if not, whether the use is planned in the future. The results in Table 3 show that 414 out of 671 participants use messenger services for customer interaction (61.7%) and that about half of the participants (340; 50.7%) use video chats for customer interaction. Since we conducted our survey after the first wave of the COVID-19 crisis, our results imply that many sales units still do not use such communication channels to offer remote advice. From our own experience, the user rates of video chats are even lower than reported in these statistics since these tools require digital skills of both intermediaries and customers. As mentioned in Sect. 3, our results could be subject to a selection of more digitally open-minded participants, since we carried out a computer-based web survey. A further limitation is that we only asked whether the participants use, e.g., video chats, but did not ask how often. Hence it might be that a lot of participants using video chats use them extremely rarely. Further results show that 495 (73.8%) of the sales units use CRM systems in order to record and analyze customer data in a structured manner. The participants using a CRM system were questioned in more detail: 299 (44.6%) are able to work with unstructured data (such as mails, phone calls, social media content) and 316 (47.1%) are able to generate automated recommendations for action based on portfolio analyses.

Table 4 provides information regarding the percentage of business transactions in the application of a contract, conclusion of a contract and claims management steps that are sent digitally to the insurance company. We see that the results are very similar across the three steps. Some sales units send no business transactions digitally to the insurance company, but others send every business transaction digitally. On average, between 76.8% (claims management) and 77.9% (conclusion of a contract) of business transactions are sent digitally to the insurance company. Since the median in all three steps is 90%, we see that at least half of the participants send at least 90% of their business transactions across all steps digitally to the insurance company. The median is much higher than the mean, which shows there are a lot of sales units using digital transmission to the insurance company, while quite a few sales units send hardly a business transaction digitally to the insurer.

Finally, Tables 5 and 6 present the descriptive statistics regarding the participants’ perceptions of the value added by the respective digital applications. Our results show that more than 50% of the participants find a CRM system highly or even very highly important. Participants see a higher value in messenger services than in video chats. 36.5% (messenger services) vs. 20.4% (video chats) of the participants assigned at least a ‘high’ value, while extended video chats with more advanced functionalities, such as screen or whiteboard sharing, are also seen as more valuable (30.6% with a value of ‘high’ or ‘very high’). These results might indicate that video chats alone are not deemed sufficient to replace face-to-face meetings, but video chats with extended functionalities are better able to do so. More than half of the participants (58.5%) find that risks assessments at the point of sale with a reduced number of questions add at least a value that is ‘rather high’. This finding implies that this might be a useful method to accelerate processes between sales units and insurance companies.

**Table 5:** Descriptive statistics regarding the participants’ perceptions of the added value given by the respective digital applications (absolute frequency and relative frequency in % in brackets)

Digital application	How much do you rate the added value?							
	Very low (1)	Low (2)	Rather low (3)	Neutral (4)	Rather high (5)	High (6)	Very high (7)	Unknown
CRM system	20 (3.0)	28 (4.2)	30 (4.5)	84 (12.5)	96 (14.3)	116 (17.3)	263 (39.2)	34 (5.1)
CRM system capable of working with unstructured data	8 (1.6)	14 (2.8)	17 (3.4)	89 (18.0)	112 (22.6)	97 (19.7)	133 (26.8)	25 (5.0)
CRM system able to generate automated recommendations based on portfolio analyses	6 (1.2)	17 (3.4)	26 (5.3)	90 (18.2)	107 (21.6)	123 (24.8)	112 (22.6)	14 (2.8)
Messenger services for customer interaction	30 (4.5)	45 (6.7)	66 (9.8)	139 (20.7)	122 (18.2)	121 (18.0)	124 (18.5)	24 (3.6)
Video chat for customer interaction	32 (4.8)	47 (7.0)	100 (14.9)	175 (26.1)	138 (20.6)	75 (11.2)	62 (9.2)	42 (6.3)
Extended video chats including screen/whiteboard sharing	35 (5.2)	29 (4.3)	62 (9.2)	154 (23.0)	136 (20.3)	89 (13.3)	116 (17.3)	50 (7.5)
Risk assessment at point of sale with a reduced number of questions	13 (1.9)	20 (3.0)	43 (6.4)	149 (22.2)	152 (22.7)	143 (21.3)	97 (14.5)	54 (8.0)
Digitally sent business transactions to the insurance company in the application of a contract	3 (0.4)	3 (0.4)	10 (1.5)	32 (4.8)	94 (14.0)	118 (17.6)	408 (60.8)	3 (0.4)
Digitally sent business transactions to the insurance company in the conclusion of a contract	3 (0.4)	5 (0.7)	6 (0.9)	42 (6.3)	106 (15.8)	126 (18.8)	377 (56.2)	6 (0.9)
Digitally sent business transactions to the insurance company in claims management	4 (0.6)	5 (0.7)	8 (1.2)	46 (6.9)	101 (15.1)	143 (21.3)	357 (53.3)	7 (1.0)

**Table 6:** Descriptive statistics regarding the participants’ perceptions of the added value given by the respective digital applications (measures of location and dispersion)

Digital application	How much do you rate the added value?					
	*N*	Mean	Median	Standard deviation	Minimum	Maximum
CRM system	637	5.5	6	1.7	Very low	Very high
CRM system capable of working with unstructured data	470	5.4	5	1.4	Very low	Very high
CRM system able to generate automated recommendations based on portfolio analyses	481	5.3	5	1.4	Very low	Very high
Messenger services for customer interaction	647	4.8	5	1.7	Very low	Very high
Video chat for customer interaction	629	4.3	4	1.6	Very low	Very high
Extended video chats including screen/whiteboard sharing	621	4.7	5	1.7	Very low	Very high
Risk assessment at point of sale with a reduced number of questions	617	5.0	5	1.4	Very low	Very high
Digitally sent business transactions to the insurance company in the application of a contract	668	6.3	7	1.1	Very low	Very high
Digitally sent business transactions to the insurance company in the conclusion of a contract	665	6.2	7	1.1	Very low	Very high
Digitally sent business transactions to the insurance company in claims management	664	6.2	7	1.2	Very low	Very high

The most valuable digital applications by mean and median are the digitally sent business transactions to the insurer. The vast majority of participants consider a digital interface with the insurance company in the application and conclusion of a contract, and in claims management, as very important (‘very high’ value added).

### Hypotheses analyses

#### Null Hypotheses 1 and 2

Tables 7, 8 and 9 respectively show the results of our chi-squared tests and Welch’s t‑tests regarding Null Hypothesis 1. For each digital application from our survey, we investigated whether there is a statistical difference between the group of exclusive agents and the group of independent agents/brokers regarding the use of this digital application.

**Table 7:** Use of digital applications among exclusive agents and among independent agents and brokers. This table shows the raw frequencies and expected frequencies (in brackets) for each digital application

Digital application	Exclusive agents		Independent agents/brokers	
	Yes	No	Yes	No
CRM system	237 (253.8)	99 (82.2)	251 (234.2)	59 (75.8)
CRM system capable of working with unstructured data	157 (147.7)	66 (75.3)	139 (148.3)	85 (75.7)
CRM system able to generate automated recommendations based on portfolio analyses	166 (154.8)	65 (76.2)	145 (156.2)	88 (76.8)
Messenger services for customer interaction	250 (216.3)	95 (128.7)	160 (193.7)	149 (115.3)
Video chat for customer interaction	183 (175.3)	164 (171.7)	149 (156.7)	161 (153.3)
Extended video chats including screen/whiteboard sharing	198 (172.1)	142 (167.9)	129 (154.9)	177 (151.1)
Risk assessment at point of sale with a reduced number of questions	172 (141.6)	149 (179.4)	102 (132.4)	198 (167.6)

**Table 8:** Results of the chi-squared tests regarding Null Hypothesis 1, i.e., the extent of the use of digital applications among exclusive agents is the same as among independent agents and brokers. This table shows for each digital application the value of the χ^2^ test statistic, the degrees of freedom *df,* the corresponding *p*-value (two-sided) and the effect size measured by φ

Digital application	Result of the chi-squared test^a^			
	χ^2^	*df*	*p*	φ
CRM system	9.50	1	0.002	0.121
CRM system capable of working with unstructured data	3.48	1	0.062	−0.088
CRM system able to generate automated recommendations based on portfolio analyses	4.87	1	0.027	−0.102
Messenger services for customer interaction	29.82	1	< 0.001	−0.214
Video chat for customer interaction	1.43	1	0.232	−0.047
Extended video chats including screen/whiteboard sharing	16.66	1	< 0.001	−0.161
Risk assessment at point of sale with a reduced number of questions	24.12	1	< 0.001	−0.197

^a^ 0 cells had an expected frequency greater than 5

**Table 9:** Results of Welch’s t‑tests regarding Null Hypothesis 1, i.e., the extent of the use of digital applications among exclusive agents is the same as among independent agents and brokers. This table shows for each digital application the value of the Welch’s t‑test statistic *t*, the degrees of freedom *df* the corresponding *p*-value (two-sided), the mean difference and the effect size measured by Cohen’s *d*

Digital application	Result of the Welch’s t‑test				
	*t*	*df*	*p*	*Mean* *Difference*^*a*^	*d*
Digitally sent business transactions to the insurance company in the application of a contract	−3.12	638.128	0.002	−6.66	−0.244
Digitally sent business transactions to the insurance company in the conclusion of a contract	−2.07	647.859	0.039	−4.24	−0.161
Digitally sent business transactions to the insurance company in claims management	−3.89	611.781	< 0.001	−8.56	−0.306

^*a*^* M*_independent agents brokers_ − *M*_exclusive agents_

For messenger services for customer interaction, we see in Table 7 that the raw frequency of exclusive agents using messenger services exceeds the expected value (250 vs. 216.3), whereas the raw frequency of independent agents and brokers using messenger services is lower than expected (160 vs. 193.7). Hence, the exclusive agents in our sample are more advanced in the usage of messenger services. This implies a possible relationship between the type of the sales unit and the use of messenger services for customer interaction. A chi-squared test in Table 8 shows that this relationship is significant, χ^2^(1) = 29.82, *p* < 0.001. Moreover, the effect size is rather low due to a φ = −0.214 (Cohen 1988). For most of the other digital applications, the exclusive agents use digital technologies in a more pronounced way than independent agents and brokers (Table 7) and the relationship is again highly significant (Table 8).

The results in Table 9 are similar. Here, we conducted Welch’s t‑tests regarding digital transmission to the insurance company in the steps of application and conclusion of a contract, and in claims management. In all three steps, there is a statistically significant difference between the percentage of digitally sent business transactions for the group of exclusive agents and the group of independent agents and brokers, with the mean percentage being higher for exclusive agents. For example, the mean percentage of digitally sent applications is 6.66 percentage points higher for exclusive agents, *t*(638.128) = −3.12, *p* = 0.002, *d* = −0.244.

Based on the results shown in Tables 7, 8 and 9, we can clearly reject Null Hypothesis 1. Compared with independent agents and brokers, exclusive agents are ahead in digital transformation, from a significantly more widespread use of digital applications. Our results indicate that the insurance companies demand and support the exclusive agents in their digital transformation, e.g., by providing appropriate tools, digital interfaces and processes to the company. Especially in the COVID-19 crisis, this support is an advantage when adapting quickly to new circumstances. The CRM system is an exception in this regard, as it is widely used by both groups in order to record and analyze customer data in a structured manner. However, the usage is even more widespread in the group of the independent agents and brokers.

The results in Tables 10 and 11 regarding Null Hypothesis 2 match the results regarding Null Hypothesis 1. We calculated Mann-Whitney U tests to determine if there were differences between exclusive agents and independent agents and brokers in the assessment of the value added by each digital application, e.g., by messenger services for customer interaction. Prior to each Mann-Whitney U test, we conducted a Kolmogorov-Smirnov test, which showed that the shapes of distribution differed between both groups (Kolmogorov-Smirnov *p* < 0.05).^2^ The results of the Mann-Whitney U tests show that, for example, there is a statistically significant difference in the assessment of the benefit from messenger services for customer interaction between exclusive agents (Mean rank = 341.7) and independent agents and brokers (Mean rank = 291.9), with* U* = 42,433, *Z* = −3.47, *p* = 0.001, *r* = −0.137.

**Table 10:** Results of the Mann-Whitney U tests regarding Null Hypothesis 2, i.e., that exclusive agents value the benefits from digital applications in the same way as independent agents and brokers. This table shows for each digital application the value of the *U*-test statistic, the value of the *Z*-test statistic, the corresponding *p*-value (two-sided) and the effect size measured by Pearson’s correlation coefficient *r*

Digital application	Result of the Mann-Whitney U test			
	*U*	*Z*	*p*	*r*
CRM system	41,279	−3.55	< 0.001	−0.142
CRM system capable of working with unstructured data	26,428	−0.33	0.740	−0.015
CRM system able to generate automated recommendations based on portfolio analyses	27,411	−0.45	0.655	−0.021
Messenger services for customer interaction	42,433	−3.47	0.001	−0.137
Video chat for customer interaction	40,936	−3.08	0.002	−0.124
Extended video chats including screen/whiteboard sharing	45,442	−0.46	0.644	−0.019
Risk assessment at point of sale with a reduced number of questions	35,963	−4.69	< 0.001	−0.190
Digitally sent business transactions to the insurance company in the application of a contract	50,267	−1.66	0.097	−0.065
Digitally sent business transactions to the insurance company in the conclusion of a contract	52,088	−0.56	0.575	−0.022
Digitally sent business transactions to the insurance company in claims management	49,799	−1.59	0.111	−0.062

**Table 11:** This table shows the mean ranks of the perceived value added by a digital application by exclusive agents and independent agents and brokers

Digital application	Exclusive agents	Independent agents and brokers
CRM system	289.4	338.5
CRM system capable of working with unstructured data	234.6	230.5
CRM system able to generate automated recommendations based on portfolio analyses	234.7	240.2
Messenger services for customer interaction	341.7	291.9
Video chat for customer interaction	288.8	332.3
Extended video chats including screen/whiteboard sharing	302.4	308.9
Risk assessment at point of sale with a reduced number of questions	334.6	269.5
Digitally sent business transactions to the insurance company in the application of a contract	339.1	317.7
Digitally sent business transactions to the insurance company in the conclusion of a contract	331.0	323.6
Digitally sent business transactions to the insurance company in claims management	337.7	316.2

Overall, Table 10 shows that exclusive agents significantly differently value five digital applications, such as messenger services and video chats for customer interaction, which is why we are able to reject Null Hypothesis 2. In the majority of these significant cases, the mean rank for the value added by the digital application is higher in the sample of exclusive agents than in the sample of independent agents and brokers (Table 11). However, for the CRM system and video chat for customer interaction, the situation is reversed.

In general, people tend to find a good more valuable when they own it (Roeckelein 2006). This might be an explanation for why exclusive agents not only use more digital applications, but also assess the value added by digital applications as higher than the assessments made by independent agents and brokers. Concerning the CRM system, this also matches the results of the extent of usage in the sales units, which is more widespread in the sample of independent agents and brokers. However, even though fewer independent agents and brokers use video chat for customer interaction, they value the benefit from video chats higher. The COVID-19 crisis increased the sense of urgency to use substitutes for face-to-face contacts with the client. Video chats are the most suitable alternative to offer advice without personal contact and the pandemic motivated the sales units to integrate video chats in their future routines (see also Table 3: 154 sales units are not yet using video chats but are planning to do so). For the other digital applications, the value gap is not so easily visible for the intermediaries if the sales units are not familiar with it. More clarification and training are necessary here if insurance sales are going to transform their approach to serve the customers’ digital expectations that come from other industries.

#### Null Hypotheses 3 and 4

Tables 12, 13 and 14 present the results of the chi-squared tests and Welch’s-t-tests regarding Null Hypothesis 3, which focuses on differences between sales units with an average age under 40 years and sales units with an average age exceeding 50 years. Again, we can reject Null Hypothesis 3.

**Table 12:** Use of digital applications among sales units with an average age under 40 years and sales units with an average age exceeding 50 years. This table shows the raw frequencies and expected frequencies (in brackets) for each digital application

Digital application	Average age < 40 years		Average age > 50 years	
	Yes	No	Yes	No
CRM system	101 (92.9)	22 (30.1)	220 (228.1)	82 (73.9)
CRM system capable of working with unstructured data	64 (64.2)	32 (31.8)	134 (133.8)	66 (66.2)
CRM system able to generate automated recommendations based on portfolio analyses	61 (65.4)	38 (33.6)	143 (138.6)	67 (71.4)
Messenger services for customer interaction	90 (77.5)	37 (49.5)	173 (185.5)	131 (118.5)
Video chat for customer interaction	84 (60.9)	43 (66.1)	123 (146.1)	182 (158.9)
Extended video chats including screen/whiteboard sharing	76 (58.6)	47 (64.4)	127 (144.4)	176 (158.6)
Risk assessment at point of sale with a reduced number of questions	66 (52.7)	56 (69.3)	113 (126.3)	179 (165.7)

**Table 13:** Results of the chi-squared tests regarding Null Hypothesis 3, i.e., that the extent of the use of digital applications among sales units with an average age under 40 years is the same as the extent of the use of sales units with an average age exceeding 50 years. This table shows for each digital application the value of the χ^2^ test statistic, the corresponding *p*-value (two-sided) and the effect size measured by φ

Digital application	Result of the chi-squared test^a^			
	χ^2^	*Df*	*p*	φ
CRM system	4.06	1	0.044	0.098
CRM system capable of working with unstructured data	0.00	1	0.955	−0.003
CRM system able to generate automated recommendations based on portfolio analyses	1.26	1	0.262	−0.064
Messenger services for customer interaction	7.34	1	0.007	0.130
Video chat for customer interaction	23.94	1	< 0.001	0.235
Extended video chats including screen/whiteboard sharing	13.85	1	< 0.001	0.180
Risk assessment at point of sale with a reduced number of questions	8.32	1	0.004	0.142

^a^ 0 cells had an expected frequency greater than 5

**Table 14:** Results of Welch’s t‑tests regarding Null Hypothesis 3, i.e., that the extent of the use of digital applications among sales units with an average age under 40 years is the same as the extent of the use of sales units with an average age exceeding 50 years. This table shows for each digital application the value of the Welch’s t‑test statistic *t*, the corresponding *p*-value (two-sided), the average difference and the effect size measured by Cohen’s *d*

Digital application	Result of the Welch’s t‑test				
	*t*	*df*	*p*	*Mean* *Difference*	*d*
Digitally sent business transactions to the insurance company in the application of a contract	1.08	257.401	0.282	2.919	0.109
Digitally sent business transactions to the insurance company in the conclusion of a contract	0.83	252.192	0.405	2.265	0.085
Digitally sent business transactions to the insurance company in claims management	1.55	281.533	0.123	4.294	0.151

^*a*^* M*_age <_ _40 years_ − *M*_age >_ _50 years_

Overall, the chi-squared tests of five out of eight digital applications in Table 13 show a significant relationship between the average age of the sales unit and the use of the digital application. In these cases, the sales units with younger sales staff use digital technologies in a more pronounced way (Table 12). For instance, Table 12 shows that the raw frequency of sales units with an average age under 40 years is higher than expected with respect to using messenger services (90 vs. 77.5) and video chats (84 vs. 60.9), whereas for sales units with an average age over 50 years the picture is reversed. The chi-squared tests in Table 13 show that this relationship is significant, i.e., for messenger services χ^2^(1) = 7.34, *p* = 0.007, φ = 0.130 and for video chat χ^2^(1) = 23.94, *p* < 0.001, φ = 0.235. However, differences between younger and older sales units regarding more advanced CRM systems using big data and artificial intelligence are not significant. One explanation might be that younger intermediaries do not have enough customers for an investment in big data or artificial intelligence to be worthwhile. Another reason may be that older intermediaries with a large customer base trust in their very experienced ‘BIO-CRM’, meaning that they memorize a lot of information about their customers and have a lot of experience in interpreting this information.

However, the results of the Welch’s t‑tests in Table 14 are not significant. Even though young sales units send slightly more business transactions digitally to insurance companies on average, there is no statistically significant difference between the percentage of digitally sent business transactions of the group with an average age under 40 years and the group with an average age exceeding 50 years. For instance, for digitally sent applications, *t*(257.401) = 1.08, *p* = 0.282, *d* = 0.109. Hence, the differences in digital usage are here vanishing between generations. An explanation for this might be that agencies and brokers with an average age exceeding 50 years often have an efficient back office that digitizes analogue documents and sends them digitally to the insurance company. In contrast to the digital applications in Tables 12 and 13, in this case, intermediaries do not have to change their familiar workflows and are still able to meet the digital expectations of insurance companies.

The results in Tables 15 and 16 allow us to clearly reject Null Hypothesis 4. Again, we calculated a Mann-Whitney U test to determine for each digital application if there were differences in the assessment of the value added between sales units with an average age under 40 years and sales units with an average age exceeding 50 years. Kolmogorov-Smirnov tests conducted prior to each Mann-Whitney U test state that the shapes of the distributions differed between both groups (Kolmogorov-Smirnov *p* < 0.05). The results then show that sales units with an average age under 40 years value all digital applications higher than sales units with an average age exceeding 50 years; this difference is statistically significant in nine out of 11 digital applications. For instance, regarding the assessment of the value added by video chats for customer interaction, there is a statistically significant difference between sales units with an average age under 40 years (mean rank = 233.64) and sales units with an average age exceeding 50 years (mean rank = 193.72), *U* = 14,080, *Z* = −3.17, *p* = 0.002, *r* = −0.157.

**Table 15:** Results of the Mann-Whitney U tests regarding Null Hypothesis 4, i.e., that sales units with an average age under 40 years value the benefits from digital applications in the same way as sales units with an average age exceeding 50 years. This table shows for each digital application the value of the *U*-test statistic, the value of the *Z*-test statistic, the corresponding *p*-value (two-sided) and the effect size measured by Pearson’s correlation coefficient *r*

Digital application	Result of the Mann-Whitney U test			
	*U*	*Z*	*p*	*r*
CRM system	14,479	−2.98	0.003	−0.146
CRM system capable of working with unstructured data	9478	−0.66	0.507	−0.038
CRM system able to generate automated recommendations based on portfolio analyses	8758	−2.09	0.037	−0.119
Messenger services for customer interaction	14,930	−2.95	0.003	−0.144
Video chat for customer interaction	14,080	−3.17	0.002	−0.157
Extended video chats including screen/whiteboard sharing	13,027	−3.56	< 0.001	−0.178
Risk assessment at point of sale with a reduced number of questions	12,706	−3.89	< 0.001	−0.195
Digitally sent business transactions to the insurance company in the application of a contract	17,345	−2.00	0.046	−0.096
Digitally sent business transactions to the insurance company in the conclusion of a contract	17,141	−1.99	0.046	−0.096
Digitally sent business transactions to the insurance company in claims management	17,183	−1.85	0.064	−0.090

**Table 16:** This table shows the mean ranks of the perceived value added by a digital application by sales units with an average age under 40 years and sales units with an average age exceeding 50 years

Digital application	Average age < 40 years	Average age > 50 years
CRM system	233.84	196.75
CRM system capable of working with unstructured data	156.77	149.79
CRM system able to generate automated recommendations based on portfolio analyses	169.71	147.51
Messenger services for customer interaction	234.56	197.30
Video chat for customer interaction	233.64	193.72
Extended video chats including screen/whiteboard sharing	231.53	187.36
Risk assessment at point of sale with a reduced number of questions	234.23	186.22
Digitally sent business transactions to the insurance company in the application of a contract	233.43	210.18
Digitally sent business transactions to the insurance company in the conclusion of a contract	233.46	209.51
Digitally sent business transactions to the insurance company in claims management	230.70	208.40

Even though during the COVID-19 crisis, older generations began to evolve a digital mindset, in which they use more digital tools and channels in everyday life, the sales units with a higher average age are less open to and less convinced by digital applications in insurance sales. Consequently, digitalization in insurance sales is still age-related.

## Discussion

### Recommendations for action

Customer experiences in various industries raise customer expectations regarding convenience and speed of customer service—requirements accelerated by COVID-19 that do not stop at the insurance industry and its sales units. However, the results of our survey imply that many traditional sales units still do not use and appreciate digital applications after the first wave of the COVID-19 crisis. To initiate a digital change in the sales process the insurer should provide and promote easy-to-use, effective and safe tools. Besides the customers’ needs the insurer must also consider the feedback of insurance intermediaries since these tools must meet the expectations and skills of both customers and intermediaries. Here, customer and agent satisfaction go hand in hand as strategic goals.

The results of our survey show e.g. that the insurance intermediaries do not see video chats alone as effective enough to replace face-to-face meetings and that more advanced functionalities, such as screen or whiteboard sharing, are needed to provide a suitable alternative. Moreover, the sales units consider the digital interface with the insurance company in application and conclusion of a contract, and in claims management as most valuable which is underlined by the high average user rates. But there are still some sales units left who send no business transactions digitally. To build on this success, but also to convince the remaining intermediaries the insurance company should improve and advertise these digital interfaces extensively. The digitally sent business transactions are the basis for automating the subsequent processes in insurance administrations. If the insurer makes significant progress in the digitalization of these operational processes, the benefit in speed can provide a self-reinforcing incentive for a digital change in sales and finally lead to customer excellence for good service.

Overall, our results indicate differences in digital usage and mindset by age and by sales channels. On average, younger intermediaries are more open-minded towards digital applications. Hence, intergenerational knowledge transfer should be enforced and clarification as well as training are needed. Additionally, the exclusive agents use significantly more digital applications than independent agents and brokers. Since the insurers support the exclusive sales units, e.g., by providing appropriate tools and digital processes to the company, the exclusive agents can adapt more easily to new circumstances. This support should reach the independent agents and broker in a similar manner. However, since these intermediaries cooperate with several insurance companies, industry standards should be established. By standardizing and optimizing business processes, different systems can be connected more efficiently. This, however, is due to the high number of insurers, independent agents and brokers in Germany challenging and, therefore, our results emphasize the importance of activities of, e.g., BiPRO e. V. (BiPRO e. V. 2021).

### Limitations and further research

As mentioned in Sect. 3, our results could be subject to a selection bias due to using a web survey design. More digitally open-minded intermediaries might be over-represented because they use the internet more extensively and, therefore, are more likely to participate. Hence, participants with higher levels of digital affinity are more likely to select themselves for the survey (Bethlehem 2010), which is why we rather overestimate the progress of the digital transformation in traditional insurance sales.

A further limitation is that the survey only asked whether the intermediaries use a digital tool, e.g., video chats, but did not ask how often, and did not capture the reasons for usage of the various tools. Moreover, we did not investigate the revenue generated by using digital tools and did not ask with respect to the percentage of customers that are actually able and interested in digital consultation. Future research in this regard could provide further valuable insights for the insurer designing the best strategy for the digital change in insurance sales.

Finally, further research could also focus on the differences in digital usage and mindset by business line. Since life and health insurers provide rather consulting-intensive products, more customers might prefer face-to-face meetings in a traditional way (see the millennials study by NÜRNBERGER Versicherung and Frankfurt Business NÜRNBERGER Versicherung and Frankfurt Business Media 2018) implying a potentially less digital sales process.

## Conclusion

In this article, we aimed to investigate the status quo of digital transformation in traditional insurance sales as well as the traditional intermediaries’ attitude towards digital applications. For this purpose, we conducted a survey in July 2020, in which 671 traditional insurance intermediaries from Germany participated. Our questions addressed a range of digital applications from CRM systems to messenger services and video chats, as well as the digital interface with insurance companies.

One main result of our survey shows that even though we conducted our survey after the first wave of the COVID-19 crisis, many traditional sales units still do not use and appreciate messenger services or video chats for customer interaction, even though these tools are the most suitable alternatives to offer advice without personal contact. However, participants see a higher value in video chats with more advanced functionalities, such as screen or whiteboard sharing. Hence, video chats alone are not seen as effective enough to replace face-to-face meetings, while video chats with more extended functionalities are more promising. A reason for these results might be a less open-minded attitude of insurance intermediaries regarding digital applications. However, a second reason might be that customers themselves still have reservations about digital applications. Easy-to-use and safe tools could remedy this problem and improve the sales process when face-to-face meetings are not possible or convenient. Overall, our results reveal that digital transformation in traditional insurance sales in general is not yet particularly advanced.

Finally, we analyzed influencing factors of sales units regarding the status quo of digital transformation. We developed four Null Hypotheses regarding differences in the status quo of digital transformation as well as the perceived benefit from it between certain subgroups. We hypothesized that there are differences between exclusive agents and independent agents/brokers, as well as between sales units with an average age under 40 years compared with sales units with an average age exceeding 50 years.

Applying chi-squared tests, Welch’s t‑tests and Mann-Whitney U tests, we found statistically significant differences between all of these groups regarding the status quo as well as the attitude towards digital transformation, allowing us to reject all four Null Hypotheses. Our results show that exclusive agents use significantly more digital applications than independent agents and brokers, and also have a more positive attitude towards digital transformation. While insurance companies accelerate their digital transformation, they motivate their exclusive agents and equip them with digital infrastructure. Hence, insurers demanding and supporting their sales staff generate an advantage in digital transformation for the exclusive agencies. Furthermore, our analyses provide insight that sales units with an average age under 40 years are further advanced in their digital transformation than sales units with an average age exceeding 50 years. We can show that the younger intermediaries place significantly higher value on the benefit of digital applications. Agencies and brokers with an older sales force often have efficient back offices supporting digitalization and the COVID-19 pandemic is changing how all generations live—e.g., habits in communication and commerce—but our results still observe age differences in digital usage and mindset.

Overall, the insurance industry should prepare their traditional sales forces to offer customer experiences that are already common in other industries. To accelerate the digital transformation in the traditional sales force the insurer should consider the feedback and needs of both customers and insurance intermediaries. More clarification and training are needed. It is important for the insurers to improve the existing traditional distribution channels by using digital tools such as video chat and instant messaging, and by accelerating processes via digitalization regarding the correspondence with the insurer and the subsequent operational processes in insurance administration. To consider independent agents and brokers, too, a standardization and optimization of business processes in the insurance industry is needed as well. Altogether, this would combine the strength of personal customer consultations with the strength of digitalization. In this way, potential can be leveraged both in the interaction with customers and in connection with efficiency gains. Finally, further research is needed, e.g. regarding the reasons of the intermediaries deciding to use or not to use digital tools, which would provide valuable insights for the insurer’s digital sales strategy.

## Footnotes

1 Based on German trade regulations (German Trade Regulations 2020), exclusive agents sell insurance contracts from one specific insurance company (Section 34d, Paragraph 7, Clause 1, German Trade Regulations), while independent agents and independent brokers are able to sell insurance contracts from more insurance companies (Section 34d, Paragraph 1, German Trade Regulations). In contrast to independent brokers, exclusive and independent agents act on behalf of insurance companies (Section 34d, Paragraph 1, German Trade Regulations). Independent brokers act on behalf of the customer (Section 34d, Paragraph 1, German Trade Regulations).

2 If the shapes of the distributions differ between both respective groups, it is not possible to interpret the Mann-Whitney U test as a test of medians but as a test of stochastic ordering of the samples (Kolmogorov 1933; Mann and Whitney 1947; Smirnov 1948; Hart 2001; Divine et al. 2018).
